# Economic burden of lung cancer in Turkey: a cost of illness study from payer perspective

**DOI:** 10.1186/s13561-021-00322-2

**Published:** 2021-06-26

**Authors:** Irfan Cicin, Ergun Oksuz, Nuri Karadurmus, Simten Malhan, Mahmut Gumus, Ulku Yilmaz, Levent Cansever, Halit Cinarka, Erdogan Cetinkaya, Murat Kiyik, Ahmet Ozet, Lung Cancer Report Study Group Turkey, Irfan Cicin, Ergun Oksuz, Nuri Karadurmus, Simten Malhan, Mahmut Gumus, Ulku Yilmaz, Levent Cansever, Halit Cinarka, Erdogan Cetinkaya, Murat Kiyik, Ahmet Ozet, on behalf of the Turkish Society of Lung Cancer, Lung Health and Intensive Care Association, University of Health Sciences, Turkish Society of Medical Oncology, National Cancer Institute, Turkish Respiratory Society

**Affiliations:** 1 Department of Medical Oncology, Faculty of Medicine, Trakya University, Edirne, Turkey; 2 Department of Family Medicine, Faculty of Medicine, Baskent University, Baglica Kampusu 06770, Etimesgut, Ankara, Turkey; 3 Gulhane Training and Research Hospital, Ankara, Turkey; 4 Faculty of Health Sciences, Baskent University, Ankara, Turkey; 5 Faculty of Medicine, Istanbul Medeniyet University, Istanbul, Turkey; 6 University of Health Sciences, Ataturk Chest Diseases and Thoracic Surgery Training and Research Hospital, Ankara, Turkey; 7 Yedikule Chest Disease and Thoracic Surgery Health Application and Research Center, University Of Health Sciences, Istanbul, Turkey; 8 Faculty of Medicine, Gazi University, Ankara, Turkey

**Keywords:** Lung cancer, Practice patterns, Cost of illness, Direct costs, Indirect costs, Economic burden, Turkey

## Abstract

**Background:**

This study was designed to estimate economic burden of lung cancer in Turkey from payer perspective based on expert panel opinion on practice patterns in clinical practice.

**Methods:**

In this cost of illness study, direct medical cost was calculated based on cost items related to outpatient visits, laboratory and radiological tests, hospitalizations/interventions, drug treatment, adverse events and metastasis. Indirect cost was calculated based on lost productivity due to early retirement, morbidity and premature death resulting from the illness, the value of lost productivity due to time spent by family caregivers and cost of formal caregivers.

**Results:**

Cost analysis revealed the total per patient annual direct medical cost for small cell lung cancer to be €8772), for non-small-cell lung cancer to be €10,167. Total annual direct medical cost was €497.9 million, total annual indirect medical cost was €1.1 billion and total economic burden of lung cancer was €1.6 billion. Hospitalization/interventions (41%) and indirect costs (68.6%) were the major cost drivers for total direct costs and the overall economic burden of lung cancer, respectively.

**Conclusions:**

Our findings indicate per patient direct medical costs of small cell lung cancer and non-small-cell lung cancer to be substantial and comparable, indicating the substantial economic burden of lung cancer in terms of both direct and indirect costs. Our findings indicate that hospitalization/interventions cost item and indirect costs were the major cost drivers for total direct costs and the overall economic burden of lung cancer, respectively. Our findings emphasize the potential role of improved cancer prevention and early diagnosis strategies, by enabling cost savings related to drug treatment and metastasis management cost items, in sustainability of cancer treatments.

**Supplementary Information:**

The online version contains supplementary material available at 10.1186/s13561-021-00322-2.

## Background

The lung cancer accounts for ~ 12% of the total new cancer cases diagnosed each year worldwide and nearly 20% of total cancer deaths [1]. Consistent with the global data, lung cancer remains the most commonly diagnosed cancer with overall prevalence of 17.3% and the leading cause of cancer mortality responsible for 23.9% of all cancer-related deaths in Turkey [1, 2].

Overall, 85 and 15% of new lung cancer diagnoses refer to non-small-cell lung cancer (NSCLC) and small cell lung cancer (SCLC), respectively [3].

Lung cancer has been associated with a substantial economic impact in terms of both direct and indirect costs, representing 15 to 23% of the total cancer-related losses [4–7]. Besides the continuous rise in lung cancer costs, a more rapid escalation is expected in the near future, due to recent emergence of new diagnostic and therapeutic interventions that may be adopted as standards of care in lung cancer management [6, 8].

Although the economic burden of lung cancer has been addressed by a relatively large amount of research, most of the studies have focused on NSCLC and direct costs related to the chemotherapy [9–11]. Accordingly, the available data are limited regarding the reliable estimates of the economic burden of lung cancer as well as the sufficient information on the drivers of the costs [9–11]. Moreover, the economic burden of lung cancer differs across countries depending on the economic development level, healthcare systems and purchasing power [12, 13]. This emphasizes the need for conducting comprehensive cost of illness studies at national level to determine the actual economic burden of the disease and to decide the areas to be improved in order to efficiently manage treatment costs and to enable better healthcare resources allocation [14–16].

Introduction of new generation and targeted treatments and immunotherapies has enabled significant improvements in treatment outcomes among lung cancer patients. However, high treatment costs can challenge the reimbursement systems with considerable limitations in public funding and availability of approved drugs to all patients due to budgetary constraints. In this regard, cost estimation of the lung cancer management based on the currently available treatment approaches may provide concrete data, contributing to resource assessments during transition to the rapidly changing treatment alternatives. However, no studies to date have addressed the total economic burden of lung cancer in Turkey in relation to combined analysis of direct and indirect costs. This cost of illness study was therefore designed to determine economic burden of lung cancer in Turkey from payer perspective using cost-of-illness method and in relation to NSCLC and SCLC subtypes.

## Methods

### Design

In this cost of illness study, per patient annual direct and indirect medical costs for the management of lung cancer as well as the total disease burden in Turkey was determined, based on national demographic/health data and literature data [2, 17–22] and/or expert panel opinion on practice patterns in clinical practice. The expert panel comprised scientific board members of The Turkish Society of Lung Cancer, The Lung Health and Intensive Care Association, The University of Health Sciences, The Turkish Society of Medical Oncology, The National Cancer Institute and The Turkish Respiratory Society. Overall, 11 experts with at least 15 years of experience in chest diseases and medical oncology were informed about the study via the above mentioned scientific societies and then participated in the four consecutive meetings to achieve the proposed consensus. The entire process including conduction of expert meetings and preparation of the consensus document was managed and validated by the four key opinion leaders (Irfan Cicin, Ergun Oksuz, Nuri Karadurmus and Simten Malhan).

### Data on real-life clinical practice

Data on real-life practice patterns in the management of lung cancer in Turkey including outpatient clinic admission rates, laboratory and radiological investigations, selected medications, hospitalizations/interventions, adverse events and metastasis were based on expert panel consensus.

### Cost analysis

Direct medical cost was calculated based on the cost items related to outpatient visits, laboratory and radiological tests, hospitalizations/interventions, drug treatment and management of drug-related adverse events and metastasis. Indirect medical cost was calculated based on the lost productivity due to early retirement, morbidity and premature death resulting from the illness, the lost productivity due to time spent by the family caregivers and visitors attending patient and cost of the formal caregivers.

The average per patient direct medical costs were calculated based on cost items including outpatient visits, laboratory and radiological tests, hospitalizations/interventions, drug treatment, adverse events and metastasis from payer perspective (only direct medical costs using prices of the public payer “Social Security Institution (SSI)” in Turkey), using cost of illness method developed by WHO [23, 24]. For drugs, retail prices from the updated price list and the updated institution discount list of SSI for 2018 were taken into account in calculation of the unit costs [25, 26]. Costs related to the diagnostic tests were calculated considering the Health Implementation Notification by SSI [23]. Physician visits costs were calculated using the unit prices based on the same SSI notification [23]. Salaries and labor force of healthcare staff giving service to lung cancer patients was provided from the Healthcare Organization Questionnaire composed of Staff Inventory Form and Information Form on the Labor Force Spent during an intervention filled for each study center. Hospitalization/interventions costs were calculated using the unit prices based on Healthcare Organization Price List in Health Practice Declaration and Treatment Assist Practice Declaration. Indirect costs comprised the value of lost productivity due to early retirement, morbidity and premature death resulting from the illness, the value of lost productivity due to time spent by family caregivers and cost of formal caregiver [27] and calculated based on the minimum income ($501 /month), retirement pension ($312/month) and disability retirement pension ($204 /month) determined by the Ministry of Family, Labor and Social Services [28] as well as the average formal caregiver pension ($224/month) calculated from average monthly salaries applied in 10 different provinces across Turkey.

The average values were considered to be 61 years for patient age, 52 years for retirement age and 78 years for life expectancy. The total number of lung cancer patients in Turkey was estimated to be 50.000 based on Turkish Ministry of Health General Directorate of Public Health [17], consisting of patients with SCLC (15%) and those with NSCLC (85%) based on expert panel opinion.

Monetary results were converted by using 5.555 €/TL July 2018 exchange rate. Direct non-medical costs of different origin (e.g. transfers of patient and caregivers for examinations and/or hospitalization, home care, etc.) and intangible costs were not included in the cost analysis.

### Statistical analysis

Descriptive statistics were used to summarize the results on practice patterns for the lung cancer management. Expenses related to the management of lung cancer were the main cost-analysis related parameter of the study. The cost model was based on the following equation: “Cost = ∑ (Frequency; %) X (Unit price; TL) X (patient ratio; %)”.

## Results

### Outpatient admission cost item

Overall, outpatient admission was estimated to occur at Medical Oncology, Chest Diseases, Thoracic Surgery and General Surgery outpatient clinics in 100.0% of patients, at Family Medicine outpatient clinics in 80% of patients, at Neurosurgery outpatient clinics in 40% of patients, at Neurology outpatient clinics in 15% of patients and at Interventional Radiology outpatient clinics in 15% of patients. SCLC and NSCLC patients were estimated to receive Medical Oncology outpatient-care for 12 times per patient per year and 18 times per patient year, respectively. Overall, 9.4% of SCLC patients and 34.0% of NSCLC patients were estimated to admit Emergency outpatient clinic, for twice per patient per year and for 8 times per patient per year, respectively (Table 1).

**Table 1:** Outpatient admission cost item: clinical practice, unit costs and total cost

Outpatient admissions	Annual admission rate (%)		Annual visit # per patient		Unit cost per admission (€)	Total cost (€)
	SCLC	NSCLC	SCLC	NSCLC		
Medical Oncology	100	100	12	18	6.62	198.65
Thoracic Surgery	100	100	10	10	7.54	150.90
Chest Diseases	100	100	19	19	7.49	285
Neurosurgery	40	40	1	1	8.10	16.39
Neurology	15	15	2	2	7.67	6.48
General Surgery	100	100	1	1	8.19	135.33
Emergency^a^	9.35	34	2	8	43.8	4.60
Family Medicine	80	80	2	2	6.55	20.50
Interventional Radiology	15	15	2	2	6.62	3.97
**Per patient outpatient admission costs (€)**			**SCLC**		Total	**338.93**
					Weighed (15%)	50.84
			**NSCLC**		Total	**489.55**
					Weighed (85%)	416.12

^a^excluding intervention, equipment and day-time bed cost

Based on unit costs, total per patient annual cost related to outpatient admissions was calculated to be €338.93 for SCLC and to be €489.55 for NSCLC (Table 1).

### Laboratory and radiological tests cost item

For both SCLC and NSCLC patients, the laboratory tests were considered to be performed at frequencies provided in Table 2, based on panel consensus.

**Table 2:** Laboratory and radiological tests cost item: clinical practice, unit and total cost

Laboratory/radiological tests	Annual rate (%)		Annual test # per patient	Unit cost (€)	Total (€)
	SCLC	NSCLC			
Liver function tests	100	100	1	Included in the package	
Kidney function tests	100	100	1		
Hemogram	100	100	1		
Complete urinalysis	100	100	1		
Erythrocyte sedimentation rate	100	100	1		
C-reactive protein	100	100	1		
Peripheral smear	100	100	1		
Protein	100	100	1		
Sputum smear	20.77	20.77			
Fasting blood glucose	100	100	1		
Lipid panel	100	100	1		
Sputum culture	12.2	12.2	1		
Electrocardiography	100	100	1		
Pulmonary function test	100	100	1		
PA chest X-ray	100	100	1		
Upper abdominal CT	100	100	1	9.91	19.82
Brain MRI	50	50	1	11.71	23.4
Lung MRI	2.5	2.5	1	11.71	0.58
Bone scintigraphy	15	15	1	14.59	4.37
Lung scintigraphy	19	19	1	24.02	9.12
Positron emission tomography	100	100	1	185.58	371.17
Bronchoscopy	90	90	1	31.51	67.08
Endobronchial ultrasonography	25	25	1	21.36	10.68
Lymph node fine needle aspiration biopsy	15	15	1	62.01	18.60
Lung fine needle aspiration biopsy	35	35	1	63.30	44.31
Thoracentesis	15	15	1	60.68	18.20
Pleural biopsy	5	5	1	40.06	4.00
Endoscopy	5	5	1	23.24	2.32
Colonoscopy	5	5	1	42.21	4.22
Mutation analysis	22.4	22.4	1	218.05	48.84
**Per patient laboratory and radiological tests cost (€)**			**SCLC**		**316.42**
			**NSCLC**		**367.24**

Based on unit costs, total per patient annual cost related to laboratory and radiological tests was calculated to be €316.42 for SCLC and to be €367.24 for NSCLC (Table 2).

### Hospitalizations/interventions cost item

Based on expert opinion, during ICU stay associated with surgery, ventilator support, vasopressor treatment and dialysis comprised the major healthcare resource utilization items. Overall, re-admission to ICU and premature death rates were considered to be 56 and 28%, respectively [18]. However, since surgery and complications during ICU stay is included in the surgery package as per Health Implementation Notification by SSI, they were not considered as cost items. Premature death was included in indirect cost calculations.

Hospitalizations were considered to occur 12 days in SCLC patients and 14 days in NSCLC patients per year at ward and re-hospitalization due to recurrence was estimated to occur in 8% for additional 10 days for both groups. The surgical procedures were considered to include pneumonectomy, segmentectomy, exploration thoracotomy and lobectomy/ bilobectomy/sleeve lobectomy and combinations. Overall, the need for radiotherapy was considered to occur in 10% of patients, while the adjuvant radiotherapy was considered to be required by 4.2% of patients along with the repeated radiotherapy in 15% of patients with NSCLC. Best supportive care was considered to be applied in 10% of patients.

Based on unit costs, total per patient annual cost related to hospitalizations/interventions was calculated to be €4116.98 for SCLC and to be €4028.75 for NSCLC (Table 3).

**Table 3:** Hospitalization/interventions cost item: clinical practice, unit costs and total cost

	Annual #of hospitalization/ interventions	Rate (%)	LOS per admission (days)	Unit daily cost (€)	Total (€)
**SCLC**					
First	1	100.0	12	24.50	293.95
Re-hospitalization	1	8.0	10	24.50	19.60
Lobectomy	1	55.0	Included in the package	1,649.0	906.95
Bi-lobectomy	1	55.0		3,298.02	1,813.91
Pneumonectomy	1	15.0		2,446.85	367.03
Sub-lobar resection	1	4.0		324.50	12.98
Exploratory thoracotomy	1	4.0		207.57	8.30
Transthoracic surgery resection	1	10.0		3,215.86	321.59
Mediastinoscopy	1	65.0		92.97	60.43
Radiotherapy	1	10.0		897.48	89.75
Blood transfusion	1	10.0		18.40	1.84
BSC	1	10.0		691.13	69.11
Radiotherapy-adjuvant	1	4.2		897.49	37.69
Radiotherapy	1	15.0		897.49	157.29
**Per patient hospitalization cost (€)**					**4,116.98**
**NSCLC**					**€**
First	1	100.0	14	24.50	342.95
Re-hospitalization	1	8.0	10	24.50	19.60
Lobectomy	1	55.0	Included in the package	1,649.0	906.95
Bi-lobectomy	1	55.0		3,298.02	1,813.91
Pneumonectomy	1	15.0		2,446.85	367.03
Sub-lobar resection	1	4.0		324.50	12.98
Exploratory thoracotomy	1	4.0		207.57	8.30
Transthoracic surgery resection	1	10.0		3,215.86	321.59
Mediastinoscopy	1	65.0		92.97	60.43
Radiotherapy	1	10.0		897.48	89.75
Blood transfusion	1	10.0		18.40	1.84
BSC	1	10.0		691.13	69.11
Radiotherapy-adjuvant	1	4.2		897.49	37.69
**Per patient hospitalization cost (€)**					**4,028.75**

*LOS* Length of hospital stay, *BSC* Best supportive care

### Drug treatment cost item

The first-line, second-line and third-line therapy rates were considered to be 100, 45 and 10% for SCLC patients and to be 74, 20 and 10% for NSCLC patients, while 25% of patients were considered to have poor performance with transition to second-line therapy For NSCLC patients, 20% of patients were considered to receive first-line therapy after adjuvant/neo-adjuvant therapy (Table 4, Supplementary Table 1).

**Table 4:** Drug treatment, drug-related adverse event and metastasis cost items: clinical practice, unit costs and total cost

**Drug treatments cost item**		**Prescription (%)**			
		**SCLC**		**NSCLC**	
**Chemotherapeutics**					
Adjuvant-Neoadjuvant		–		20	
Advanced first-line therapy		100		74	
*Poor performance*		25		25	
Advanced second-line therapy		45		20	
Advanced third-line therapy		10		10	
**Other drugs**		**SCLC**	**NSCLC**	**Total (€)**	
Analgesics		85	85	243.74	
Steroids		45	45	11.16	
Bisphosphonates		20	20	648.96	
Nutrition (3-month)		100	100	320.07	
G-CSF		2.5	2.5	841.94	
**Per patient drug treatment cost (€)**		**SCLC**		**1,484.19**	
		**NSCLC**		**2,765.58**	
**Adverse events cost item**		**Annual rate (%)**		**Unit cost (€)**	**Total (€)**
		**SCLC**	**NSCLC**		
Rash		2	2	90.5	1.81
Febrile Neutropenia		4	4	5,019.9	200.79
Anemia		50	50	144.3	72.16
Nausea/Vomiting		100	100	77.6	77.60
Diarrhea		15	15	29.99	5.40
Constipation		70	70	18.4	12.89
Pulmonary Toxicity		25	25	635.51	133.87
Fatigue		75	75	194.16	145.62
Hypoalbuminemia		10	10	281.67	28.16
Neutropenia		6	6	983.77	59
Deep Vein Thrombosis		10	10	586.19	58.62
Infection		50	50	11.75	5.9
Anorexia		50	50	364.5	182.23
Thrombocytopenia		5	5	162.34	8
Edema		10	10	26	2.60
**Per patient drug-related adverse event cost (€)**					**999.57**
**Metastasis cost item**		**Annual rate (%)**		**Unit cost (€)**	**Total (€)**
		**SCLC**	**NSCLC**		
Central Nervous System	diagnosis	23	23	1,811.06	93.72
	follow up	40	40	1,811.06	93.72
Bone		32.5	32.5	1,253.87	407.51
Liver		12.5	12.5	4,058.71	507.34
Pleura		14.3	14.3	102.02	14.59
Kidney		15	15	1,252.42	21.29
**Per patient metastasis cost (€)**					**1,516.40**

*G-CSF* Granulocyte- Colony Stimulating Factor

Apart from chemotherapy, SCLC and NSCLC patients were considered to receive analgesic, steroid, bisphosphonate, 3-month nutritional support and G-CSF therapy (Table 4).

Based on prescription rates in Turkey, maintenance doses and annual dose and unit cost per box (chemotherapy and intravenous treatment) or per tablet (oral treatments) for each drug regimen, total per patient annual cost related to drug treatment was calculated to be €1484.19 for SCLC and to be €2765.58 for NSCLC (Table 4).

### Drug-related adverse events cost item

Similarly in SCLC and NSCLC patients, adverse event costs were calculated based on treatment algorithm and unit cost per box depending on posology [19] (Table 4).

Based on unit costs, total per patient annual cost related to drug-related adverse events was calculated to be €999.57 (Table 4).

### Metastasis cost item

Based on expert panel consensus along with the literature data, metastasis was considered to develop in 70–75% of SCLC patients and 60% of NSCLC patients and to involve central nervous system, bone, lung, kidney, pleura, liver and adrenal gland. Central nervous system metastases were considered to present in 23% of patients at the time of diagnosis and to develop during follow up in 40% of patients [20–22] (Table 4).

Based on entire direct cost items related to metastasis management, total per patient annual cost related to metastasis was calculated to be €1516.40 (Table 4).

### Per patient total annual direct medical cost

Total per patient annual direct medical cost related to management of SCLC was calculated to be €8772.49 from payer perspective. For SCLC, hospitalizations/interventions (46.9%) was the major direct cost driver, as followed by metastasis treatment (17.3%) (Table 5).

**Table 5:** Per patient and total annual direct medical cost and total annual indirect -cost related to management of lung cancer

**Direct cost**	**Per patient annual cost (€)**		**Contribution to per patient cost (%)**		**Total cost (€)**	**Contribution to total cost (%)**
	**SCLC**	**NSCLC**	**SCLC**	**NSCLC**		
**Number of patients**	7,500	42,500				
**Direct cost items**						
Outpatient admission	338.94	489.56	3.8	4.8	41,328,467.61	8
Laboratory test-imaging	316.42	367.24	3.6	3.6	^a^	^a^
Hospitalization/intervention	4,116.98	4,028.75	46.9	39.6	202,099,412.37	41
Drug treatment	1,484.19	2,765.58	16.9	27.2	128,668,391.79	26
Adverse events	999.57	999.57	11.4	9.8	49,978,377.04	10
Metastasis	1,516.40	1,516.4	17.3	14.9	75,820,050.53	15
**Total direct per patient cost**	**8,772.49**	**10,167.07**				
**TOTAL DIRECT COST(€)**			**497,894,699.34**			
**Indirect cost**						
**Cost items**	**Number of persons**		**Total cost**			
**Lost productivity (patient)**						
Lost productivity due to premature death	15,000		529,306,931			
Lost productivity due to time spent in hospital	50,000		31,480,660			
Travel distance	30,000		16,633,663			
Lost productivity due to disability retirement	4,000		167,805,581			
Lost productivity due to medical reports	25,000		162,769,322			
Lost productivity due to early retirement	8,800		141,148,515			
Total	50,000		1,049,144,671			
**Lost productivity (family caregiver)**	47,500		29,906,627			
**Formal caregiver cost**	2,500		5,751,575			
**TOTAL INDIRECT COST (€)**			**1,084,802,873.6**			

^a^Included in outpatient cost item

Total per patient annual direct medical cost related to management of NSCLC was calculated to be €10,167.07 from payer perspective. For NSCLC, hospitalizations/interventions (39.6%) was the major direct cost driver, as followed by drug treatment (27.2%) (Table 5).

Based on consideration of SCLC (15%) and NSCLC (85%) rates in overall 50.000 patients, the number of patients with SCLC and NSCLC were estimated to be 7500 patients and 42.500 patients, respectively.

Accordingly, total annual direct medical cost was calculated to be €497,894,699.3 for lung cancer. Hospitalizations/interventions (€ 202,099,412.37, by 41%) was the major direct cost driver, followed by drug treatment (€128,668,391.79, by 26%) (Table 5).

### Indirect costs

Based on expert panel opinion, the rates for active employees, premature death, medical reports, disability retirement, early retirement, family caregivers, formal caregivers and travel distance to hospital were estimated. The cost of travel to hospital (patients and caregivers) form another province was calculated as 70 TL per person based on the minimum wage considered by Turkish Federation of Drivers and Automobilists [29]. The value of lost productivity due to time spent in hospital (44 days per year) was calculated for both patients and caregivers attending patient (Table 5).

The average value of lost productivity was calculated to be €1.0 billion for patients and to be €30 million for family caregivers, while the formal caregiver cost was calculated to be €5.8 million. Accordingly total annual per patient indirect cost was calculated to be €1.1 billion (Table 5).

### Total economic burden of lung cancer

Based on total annual direct medical cost and indirect cost for patients, total economic burden of lung cancer was calculated to be €1.6 billion (Table 6).

**Table 6:** Economic burden of lung cancer in Turkey

	**Direct cost**	
	**SCLC**	**NSCLC**
Number of patients	7,500	42,500
Per patient cost (€)	8,772.49	10,167.07
**Total direct cost (€)**	**497,894,699.34**	
	**Indirect cost**	
	**Number of persons**	**€**
Patient	50,000	1,049,144,671
Family caregiver	47,500	29,906,627
Formal caregiver	2,500	5,751,575
**Total indirect cost (€)**		**1,084,802,873.6**
**TOTAL LUNG CANCER BURDEN (€)**		**1,582,697,573.0**

The major cost driver was indirect costs (68.6%), while direct cost contributed to 31.4% of total disease burden.

## Discussion

Our findings related to total per patient annual direct medical cost for SCLC and NSCLC, total annual direct non-medical cost, total annual indirect medical cost, and total economic burden of lung cancer confirm the consistently reported real-world data on the substantial economic burden of lung cancer to the healthcare system [9–11].

In the current study, hospitalization/intervention was the major per-patient direct cost driver for SCLC and NSCLC and the major cost driver for the total direct costs. The drug treatment was the next largest cost driver for the total direct cost and per-patient direct cost in NSCLC, while the contribution of metastasis and drug treatment to per-patient direct cost was similarly high in SCLC.

Likewise, largest cost drivers in NSCLC patients were reported to be associated with therapies received (€12,375 France; €3694 UK) and hospitalization/emergency costs (€7706 Germany) [11], while the main cost drivers (total cost: $45,897) in lung cancer in the USA was reported as hospitalization (49.0%) and outpatient office visits (35.2%) [30]. The systemic anti-cancer medication was reported as the main cost driver that comprise 77.4% of total costs in a multinational European study [9], and noted as the key cost driver in studies from Italy (€25,859) [31] and the Netherlands (€17,463) [32].

In a study with 24,729 NSCLC patients in Canada, authors reported the overall total cost to be $1.9 billion, and indicated inpatient hospitalizations as the major cost driver as followed by outpatient visits and physician services [33]. In a past study with 66,535 lung cancer patients in Taiwan, the lifetime healthcare expenditures were reported to be $18,455 for SCLC, $20,599 for squamous cell carcinoma and $36,771 for adenocarcinoma [8]. In a past study from South Korea in lung cancer patients using a nationwide claims database, the 5-year medical expenditure per case was reported to be highest in the surgery+CTx/RTx group ($36,013), followed by the CTx/RTx ($23,134), surgery ($22,686), and supportive treatment group ($3700) [14]. In contrast, the lung cancer-related anti-cancer drug therapy was noted as the major cost driver with an average 53% share across all patients [14]. Further analysis of lifetime estimates by the same authors revealed the overall mean cost per year to be 4359 USD in surgery, 7075 USD in Surgery+CTx/RTx and 7626 USD in CTx/RTx groups [34].

Hence, our findings support the previous reports that indicated hospitalization/interventions costs as the leading direct cost driver to the total economic burden of lung cancer that ranges from 31 to 71% based on findings from several countries, as followed by drug treatment cost [35–38]. Being the third largest direct cost driver, per patient costs related to adverse event management (€1167) in the current study seems also consistent with the total mean per-patient costs associated with management of adverse events during adjuvant treatment reported in France (€1063), in Germany (€1282) and in the UK (€894) [11].

Also, our findings emphasize the larger contribution of drug treatment cost item to total direct cost in NSCLC vs. SCLC (27.2 vs. 16.9%) patients, in line with consideration of administration of adjuvant-neoadjuvant therapy in 20% of NSCLC patients. Likewise, in a European study, lower total overall direct costs in the UK (€8377) compared to France and Germany (€19,057 and €14,185 respectively) was considered to be related to a lower proportion of patients receiving adjuvant therapy (33.4% in the UK versus 61.8% in France and 51.9% in Germany) and lower costs after disease progression in the UK [11].

Indeed, an increase in healthcare expenses is considered for NSCLC and especially for patients diagnosed with adenocarcinoma due to the new treatment therapies used, including molecular targeted drugs, immunotherapies and third-generation chemotherapies) [36, 39].

Notably, histological type, disease stage, disease progression, the number of treatments received and the proportion of patients receiving adjuvant therapy have been considered significant clinical events in cost implications regarding lung cancer [10, 11, 21, 30, 36, 40]. Analysis of the Surveillance, Epidemiology, and End Results (SEER)-Medicare database for the years 1991 through 2003 in 60,231 lung cancer patients revealed the estimated direct lung cancer care costs to range from $12,411 to $16,619 in NSCLC and from $16,105 to $17,321 in SCLC [40].

In a study from USA on per patient annual costs for elderly patients with extensive-stage SCLC and metastatic NSCLC, authors reported that in relation to increased use of chemotherapy, supportive care therapies, and disease-related hospitalizations, per-patient total all-cause health care costs ($70,549 vs. $67,176), as well as total disease-related per-patient costs, were higher in extensive-stage SCLC patients ($44,167 vs. $37,932) [10]. In a past study among 2040 lung cancer patients in the USA, mean per patient monthly total costs was reported to be $6520 [30]. The authors also noted higher per patient costs in initial treatment phase vs. secondary treatment or terminal care phase along with additional $10,370 and $8779 cost increment per month in case of treatment failure in initial treatment phase and after starting the secondary and/or terminal care phase, respectively [30].

The total per-patient cost of care of advanced NSCLC in Spain was estimated to range from €11,301 to €32,754 depending on the number of treatments received [21], while data from lung cancer patients in the USA revealed a remarkable increase in the cost of treating advanced NSCLC during disease progression [41].

Notably, in the current study indirect costs (68.6%) were the major cost driver for the total economic burden of lung cancer, while direct costs accounted only for 31.4% of total disease burden. Similarly, in a methodologic review on cost of lung cancer, the authors considered lung cancer to be a costly illness with hospitalization and treatments accounting for a large part of direct costs, while the indirect costs represent a large part of the total costs [42].

In European countries, the total burden of lung cancer was estimated to be €106.4 billion with direct costs accounted for €3.35 billion of the total cost and indirect costs related to disability and premature mortality accounted for €100 billion and per patient direct cost of €11,473 [4, 6]. Other studies also revealed the hospitalization, mortality and indirect costs to account for the largest part of the direct, indirect and total costs for lung cancer, respectively [6, 15, 42, 43].

In the analysis of data derived from 2010/2011 EU National Health and Wellness Survey among relatives of patients with lung cancer in France, Germany, Italy, Spain, and the UK, the relatives providing care for a patient with lung cancer reported significantly greater work impairments and were associated with higher indirect costs (productivity losses) compared with the relatives not providing care [44]. Moreover, caregiver costs were also reported to increase with an increasing stage at diagnosis of lung cancer with a 53.9% higher economic burden than caring for a patient diagnosed at Stage IV vs. Stage I [4, 45].

Nonetheless, it should be noted that the direct comparison among different cost of illness studies is considered challenging in terms of indirect as well as direct costs, given the heterogeneity between studies regarding demographic characteristics, cost scope, observation period and the database used as well as the different socioeconomic environment, medical settings and income level between countries [5, 7, 14, 46].

Lung cancer incurs serious economic overburden in diagnosis and treatment according to the increasing number of patients, while 20% of expenditures for all types of cancer treatment are considered to be due to lung cancer [43, 47]. The overall cost of cancer care was $124.5 billion in the US in 2010, while costs specific to lung cancer accounted for $12.1 billion [48]. Providing data on the substantial contribution of hospitalization and drug treatment costs to total direct costs related to management of lung cancer in both NSCLC and SCLC patients, our findings seem to emphasize the potential role of new strategies for lung cancer that reduce hospitalizations and/or prevent or delay treatment failure in limiting the economic burden associated with the disease [30]. In this regard, sustainability of cancer treatments seems to be related not only to the proportion of healthcare budget spent on cancer but also to the improved cancer prevention and early diagnosis strategies that would enable cost savings related to drug treatment and metastasis management cost items. The new generation treatments may also offer a cost benefit by enabling an improved quality of life through a clinical course with reduced risk of toxicity, with potential projections to the overall oncological expenditures. Moreover, given that lung cancer is one of the leading causes of avoidable death, the primary focus and cost-saving strategy must be on the prevention of the disease by effective smoking cessation interventions and thus the reduction in disease burden.

The major strength of the current study seems to be an analysis of not only direct costs but also the indirect costs (loss of productivity due to the illness) in both NSCLC and SCLC patients which likely to prevent a downward bias in our estimates of the economic cost of lung cancer. However, certain limitations to this study should be considered. First, use of expert consensus based data rather than national database on practice patterns to identify direct medical costs might raise a concern with the validity and reliability of the data. Second, while a cost-of-illness study gives a perspective on the economic burden of lung cancer in a population, it does not reflect what is happening with the individual patient or family unit. Nevertheless, providing the cost estimates for management of lung cancer patients with NSCLC and SCLC subtypes in Turkey, our findings represent a valuable contribution to the literature.

## Conclusions

Our findings indicate per patient direct medical costs of both SCLC and NSCLC to be substantial and comparable and confirm the substantial economic burden of lung cancer in terms of both direct and indirect costs. Our findings indicate hospitalization/intervention and indirect costs as the major cost drivers for total direct costs and the overall economic burden of lung cancer, respectively.

In this regard, our findings emphasize the role increasing the proportion of healthcare budget reserved for lung cancer and implementation of the improved cancer prevention and early diagnosis strategies in sustainability of cancer treatments, by enabling cost savings related to drug treatment and metastasis management cost items. Moreover, consideration of indirect costs with the assessment of total productivity losses in planning cost-saving approaches seems also important to assist decision makers in the allocation of resources, while the promotion of programs aiming to reduce the incidence of lung cancer in working-age individuals seems likely to enable substantial reductions in productivity loss counts. Accordingly, health policies in lung cancer should be developed with particular emphasis on preventive healthcare strategies such as implementation of effective measures targeting smoking prevention/cessation and early diagnosis with effective and wide use of screening methods to reduce the burden of premature cancer-related mortality as well as the potential role of new generation treatment alternatives or immunotherapy in achievement of improved quality of life and cost-savings for adverse effect management, hospitalization expenses or lost work-force.

## Supplementary Information

**Additional file 1: Supplementary Table 1.** Distribution of chemotherapeutic treatments according to treatment lines.

## Data Availability

The datasets during and/or analyzed during the current study available from the corresponding author on reasonable request.

## Abbreviations

NSCLC: Non-small-cell lung cancer
MRI: Magnetic resonance imaging
SCLC: Small cell lung cancer
SSI: Social Security Institution
