# Supplementary material for: Economic burden of lung cancer in Turkey: a cost of illness study from payer perspective
Source: Health Econ Rev. 2021 Jun 26;11:22. doi: 10.1186/s13561-021-00322-2 (PMC8233643; doi:10.1186/s13561-021-00322-2)
Supplement: Supplementary file 1 — Additional file 1: Supplementary Table 1. Distribution of chemotherapeutic treatments according to treatment lines. [file 13561_2021_322_MOESM1_ESM.doc]

Supplementary Table 1. Distribution of chemotherapeutic treatments according to treatment lines

| **Therapies** | **%** |
| --- | --- |
| **Adjuvan/neo-adjuvan therapies** | |
| Cisplatin+pemetrexed | 5.0 |
| Cisplatin+gemcitabine | 5.0 |
| Carboplatin+paclitaxel | 4.0 |
| Cisplatin+vinorelbine | 2.0 |
| Cisplatin+etoposide | 1.4 |
| Cisplatin+docetaxel | 1.1 |
| Cisplatin | 0.7 |
| Carboplatin | 0.7 |
| **Advanced first-line therapies** | |
| Cisplatin+vinorelbine | 21.2 |
| Cisplatin+gemcitabine | 21.2 |
| Oral etoposide | 25.0 |
| ALK-positive-target agent | 5.0 |
| **Second-line therapies** | |
| Pemetrexed | 10.8 |
| Docetaxel | 8.2 |
| ALK-positive-target agent | 1.0 |
| **Third-line therapies** | |
| Gemcitabine | 4.8 |
| Paclitaxel | 4.8 |
